# Possible temporal relationship between SARS-CoV-2 infection and anti-NMDA receptor encephalitis: a meta-analysis

**DOI:** 10.1038/s41398-024-02831-0

**Published:** 2024-03-08

**Authors:** Veronika Vasilevska, Paul C. Guest, Michael Szardenings, Michael E. Benros, Johann Steiner

**Affiliations:** 1 Department of Psychiatry, Otto-von-Guericke-University Magdeburg, Magdeburg, Germany; 2 Laboratory of Translational Psychiatry, Otto-von-Guericke-University Magdeburg, Magdeburg, Germany; 3 Laboratory of Neuroproteomics, Department of Biochemistry and Tissue Biology, Institute of Biology, University of Campinas (UNICAMP), Campinas, Brazil; 4 Ligand Development Unit, Fraunhofer Institute of Cell Therapy and Immunology, Leipzig, Germany; 5 Copenhagen Research Centre for Mental Health, Mental Health Center Copenhagen, Copenhagen University Hospital, Hellerup, Denmark; 6 Center for Behavioral Brain Sciences (CBBS), Magdeburg, Germany; 7 Center for Health and Medical Prevention (CHaMP), Magdeburg, Germany; 8 German Center for Mental Health (DZPG), Partner Site Halle-Jena-Magdeburg, Magdeburg, Germany; 9 Center for Intervention and Research on Adaptive and Maladaptive Brain Circuits Underlying Mental Health (C-I-R-C), Halle-Jena-Magdeburg, Magdeburg, Germany

**Keywords:** Molecular neuroscience, Psychiatric disorders

## Abstract

The global impact of SARS-CoV-2 infection has raised concerns about secondary diseases beyond acute illness. This review explores the significance and potential underlying mechanisms of how SARS-CoV-2 infection might elicit an immune response targeting N-methyl-D-aspartate (NMDA) receptors, and its implications for autoimmune-driven neuropsychiatric manifestations. We identified 19 published case reports of NMDA receptor encephalitis associated with SARS-CoV-2 infection or vaccination by a systematic literature search. The significance of these reports was limited since it is not clear if a coincidental or causal relationship exists between SARS-CoV-2 infection or vaccination and manifestation of NMDA receptor encephalitis. The included studies were hampered by difficulties in establishing if these patients had pre-existing NMDA receptor antibodies which entered the brain by infection- or vaccination-associated transient blood-brain barrier leakage. In addition, four cases had comorbid ovarian teratoma, which is a known trigger for development of NMDA receptor encephalitis. Considering that billions of people have contracted COVID-19 or have been vaccinated against this virus, the publication of only 19 case reports with a possible link to NMDA receptor encephalitis, indicates that it is rare. In conclusion, these findings do not support the case that SARS-CoV-2 infection or vaccination led to an increase of existing or de novo encephalitis mediated by an autoimmune response targeting NMDA receptor function. Nevertheless, this work underscores the importance of ongoing vigilance in monitoring viral outbreaks and their potential impact on the central nervous system through basic, epidemiological and translational research.

## Introduction

### Background

Although most coronavirus-dedicated websites have stopped collecting data on COVID-19 cases caused by infection with the SARS-CoV-2 virus [1, 2], agencies such as the Institute for Health Metrics and Evaluation Model have estimated that more than half of the world population had been infected at least once, as of January 2022 [3]. In addition, a significant proportion of people worldwide have been re-infected by the virus, particularly during the more recent waves of the SARS-CoV-2 B.1.1.529 (Omicron) sub-variants [4–7]. This has been linked to the high number of mutations in the Omicron strains, which has resulted in a higher transmissibility rate and increased ability of the virus to evade the immune system and reduce vaccine efficacy [8–11]. Furthermore, 10–30% of SARS-CoV-2 cases have been found to suffer from long-term effects of infection, which has been termed post-COVID syndrome (PCS; also known as long COVID) [12–14]. According to the National Institute for Health and Care Excellence (NICE) guidelines, PCS is characterised as “signs and symptoms that develop during or after an infection consistent with COVID‑19, continue for more than 12 weeks and are not explained by an alternative diagnosis” [15]. Although the clinical manifestation is not homogeneous, PCS may occur as various overlapping symptoms and include chronic fatigue and physical complaints, as well as neurological and neuropsychiatric presentations [16, 17].

Before the advent of the vaccines, most countries in the world enacted similar strategies to reduce the prevalence of the SARS-COV-2 virus and to save lives, including different lockdown and quarantine approaches, as well as implementing social restriction policies [18–20]. Although these policies were mostly effective in reducing the infection rate, the side effect was an increase in neuropsychiatric symptoms such as stress, anxiety and depression [21–25]. For example, the Office for National Statistics in the United Kingdom estimated that there was a doubling of adults experiencing either moderate or severe depression symptoms from 10 to approximately 20% during the first year of the pandemic [26, 27]. In addition to being a response to social restriction measures, reports demonstrated that some of the neurological and psychiatric effects may have resulted directly from infection with the SARS-CoV-2 virus [28–31].

### COVID-19 infection and autoimmune outcomes

Some severe cases of SARS-CoV-2 infection or PCS have demonstrated effects on organs and tissues of the body similar to autoimmune diseases marked by the presence of autoantibodies [32–37], interstitial pneumonia with autoimmune features (IPAF) [38], antineutrophil cytoplasmic antibody (ANCA)-associated vasculitis (AAV) [39], systemic lupus erythematosus (SLE) [40], rheumatic musculoskeletal diseases [41], and neurological conditions such as Guillain-Barré syndrome (GBS) [42]. In addition, exacerbation or deterioration has been observed in patients with psychiatric disorders, including autism, schizophrenia [43], depression and anxiety disorders [44].

We and others have proposed that mimicry of the accessory, structural and non-structural proteins of the SARS-CoV-2 virus may be one of the underlying causes of autoimmune response against peripheral and central nervous system (CNS) G protein-coupled and ion channel receptors [45, 46]. In the latter study, we performed a meta-analysis and identified 8 potential cases of N-methyl-D-aspartate (NMDA) receptor encephalitis associated with SARS-CoV-2 infection [45]. This autoimmune disorder can present with neurological and psychosis-like symptoms and is diagnosed according to the clinical signs, as well as findings of brain magnetic resonance imaging (MRI), electroencephalography (EEG) and the presence of autoantibodies against NMDA receptors in blood serum, plasma or cerebrospinal fluid (CSF) [47, 48].

## Molecular mimicry and autoimmunity following SARS-CoV-2 infection

As described above, autoimmunity is common after SARS-CoV-2 infection [49], and molecular mimicry may play a crucial role in this.

### Types of molecular mimicry

We highlight three types of molecular mimicry that could cause autoimmune responses following a viral infection, as reviewed in previous publications [49–51]. The first type is caused by structural similarities between viral and host proteins. In the second type, the foreign antigen on the infectious agent contains similar epitopes to a host antigen but is different enough to induce the release of pro-inflammatory cytokines and chemokines (e.g. by antigen-presenting dendritic cells, macrophages and CD4 + T helper cells, which orchestrate the immune response). A third type of molecular mimicry is recognition of dissimilar chemical structures on separate molecules by a single antibody by chance. Cross-reactive peptide epitopes have been identified [52] or theorized [53] for SARS-CoV-2 and other coronaviruses. Given the large size of the SARS-CoV-2 proteome and the potential for severe activation of the immune response, it is likely that one or more types of mimicry are involved.

### SARS-CoV-2 structure

SARS-CoV-2 is an enveloped virus particle containing a positive-sense 30 kb RNA genome, which is stabilized by nucleocapsid proteins. This is surrounded by a membrane containing envelope, membrane proteins, and the membrane anchored spike proteins that enable the infection of host cells [54–56]. The RNA genome encodes 16 non-structural proteins which, when activated, are involved in the infection and replication processes [57, 58], as well as the spike, envelope, membrane and nucleocapsid structural proteins and 3a, 6, 7a, 7b, 8, 9b, and 10 accessory proteins [59, 60].

### SARS-CoV-2 and molecular mimicry

Viruses such as SARS-CoV-2 are well-known for having the potential to initiate inflammatory and autoimmune responses in infected individuals [61]. Related to this, a study by Yapici-Eser et al. proposed that some of the psychiatric symptoms associated with SARS-CoV-2 infections may be caused by the ability of the viral proteins to mimic host protein interactions, such as those of G-protein-coupled receptors (GPCR) and ligand-gated ion channel receptor proteins involved in neuronal signalling [46]. This included the SARS-CoV-2 non-structural proteins 8 (NSP8) and 9 (NSP9) which may mimic interactions of the host NMDA receptor NR2A and NR1 subunits, respectively. This kind of mimicry may lead to bystander T cell activation and epitope spreading, giving rise to an autoimmune reaction [62].

## Aims

In this paper, we have extended our previous meta-analysis [45] by searching the PubMed and Google Scholar databases to identify all cases reports of NMDA receptor encephalitis associated with SARS-CoV-2 infection or COVID-19 vaccination. We aimed to describe potential mechanisms on how SARS-CoV-2 infection can lead to the development or exacerbation of autoimmune NMDA receptor encephalitis and summarize a potential screening and treatment approach for this condition in acute and post-SARS-CoV-2 infected patients. This is followed by critical reflections on the evaluation of the case reports found, open questions on causality and limitations of our meta-analysis.

## Methods

**Fig. 1:**
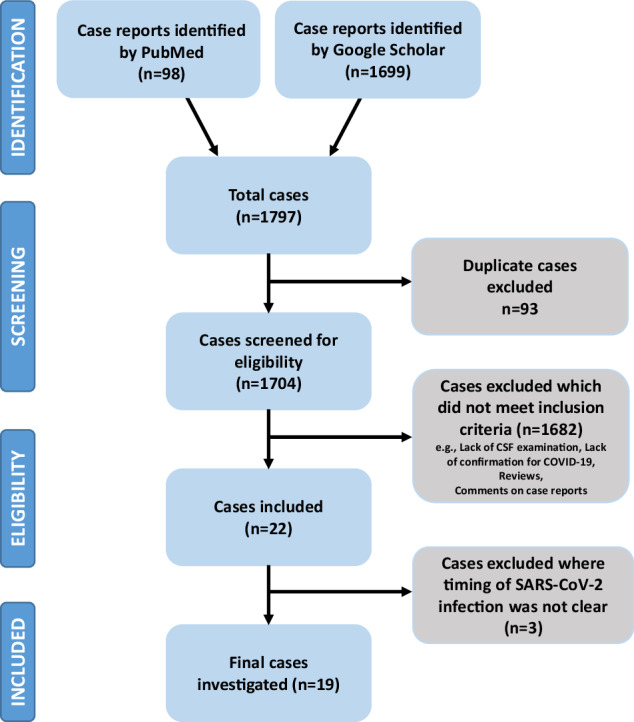
PRISMA flowchart illustrating our systematic literature search and selection process. Identification: The original database search resulted in 98 records from PubMed and 1699 records from Google Scholar (in total 1797 records). Screening: After removing 93 duplicates, there were 1704 unique citations eligible for screening of the publication content. Eligibility: In the first phase of the eligibility check, 1682 records were excluded because they did not meet the inclusion criteria (e.g. missing CSF examination, missing COVID-19 confirmation, reviews, comments on case reports). This process left 22 records to be reassessed for eligibility by a very thorough review of all available data in the full-text articles. The second stage of screening excluded 3 articles where the timing of SARS-CoV-2 infection was not clear. Included: Finally, 19 articles were included in our systematic review.

We searched PubMed and Google Scholar databases using the search terms “NMDA encephalitis” or “NMDA” and “SARS-CoV-2” or “COVID-19” to identify relevant cases. The last search was performed on May 3rd, 2023. Preferred Reporting Items for Systematic Reviews and Meta-Analyses (PRISMA, http://prisma-statement.org) were applied. The flow diagram for identification, screening, eligibility and inclusion of studies is illustrated in Fig. 1. Studies were checked for eligibility and selected by two authors (VV and PCG). Initially, many more papers were identified in Google Scholar than in PubMed. However, most of these articles did not meet the search criteria as, on close inspection, these were found to be comments on already published articles or related meta-analyses. For the final evaluation, only case or original reports published in peer-reviewed international journals were considered. For a complete review, we also included the case reports described in our previous meta-analysis [45]. Quality was assessed according to the Case Report Guidelines (CARE; https://www.care-statement.org/). Articles in languages other than English or German were translated using DeepL Translator [63]. Studies were included if cases showed SARS-CoV-2 positivity in nasopharyngeal swab, blood or CSF tests and had clinical signs of NMDA receptor encephalitis [47]. Data on patient age, medical history, hospitalization reason, respiratory status, psychiatric symptoms, brain MRI analyses, EEG readings, and blood-or CSF-based biomarkers were mined as available. Information on attempted therapies and clinical outcomes were extracted if these were indicated.

As a supplement, we checked whether the cases described fulfilled the criteria of Graus et al. [47] for definite or probable NMDA receptor encephalitis. If the publications did not contain the necessary information, the authors were contacted to obtain this data.

## Results

A search of the PubMed and Google Scholar databases led to identification of 1797 total cases of NMDA receptor encephalitis using the search terms described in the Methods section. Of these, 93 cases were excluded which appeared in duplicate articles and 1682 were removed which did not include CSF examination or confirmation for COVID-19 infection, or they were reviews or comments on case reports (Fig. 1). The 8 cases that we reported on previously were included [45], leaving 22 articles. Finally, three further cases were excluded as the timing of SARS-CoV-2 infection in these was not clear [64–66]. This left a final 19 articles which met all criteria for this meta-analysis (Table 1). The strict criteria for NMDA receptor encephalitis proposed by Graus et al. [47] were definitely met in 13 of the 19 cases, while 3 cases met the criteria for probable NMDA receptor encephalitis. In 3 cases this was unclear, as not all data were available for this assessment (see Supplementary Table).

**Table 1:** Summary of case reports (*n* = 19) which met all criteria for this meta-analysis.

Patient 1	Sanchez-Larsen et al. [67]	Female (22 years old). Known non-lesional focal right frontal lobe epilepsy since 2 years
	Reason for hospitalisation	4 focal to bilateral tonic-clonic seizures
	Respiratory (other) symptoms	COVID-19 positive (test method not mentioned) with mild flu-like symptoms Examination revealed no teratoma
	Neuropsychiatric symptoms	Severe anxiety, dysphoric mood, insomnia, motor aphasia, visual hallucination, three focal to bilateral tonic-clonic seizures within several hours, bradyphrenia, psychomotoric agitation
	Blood test	Normal
	CSF examination	White cell count 7/µl, normal protein and glucose levels. SARS-CoV-2 not mentioned. Additional virological and microbiological diagnostics negative. NMDAR-antibodies positive (Ig class and test method not mentioned).
	EEG	Frontal intermittent rhythmic delta activity, with no other relevant findings
	Brain imaging	MRI: normal (unclear if contrast agent was used)
	Therapy	Antiseizure therapy, benzodiazepines and antipsychotics administered After NMDAR encephalitis diagnosis, methylprednisolone, intravenous immunoglobulin and rituximab administered
	Course	Not mentioned
Patient 2	Valadez-Calderon et al. [79]	Male (28 years old)
	Reason for hospitalisation	Fronto-orbital syndrome characterized by catatonic symptoms
	Respiratory (other) symptoms	Mild COVID-19 (test method not mentioned)
	Neuropsychiatric symptoms	Incoherent speech, somnolence, auditory hallucinations, suicidal ideations and generalized tonic-clonic seizures, status epilepticus
	Blood test	Normal
	CSF examination	Initial CSF analysis was normal (white cell count not mentioned). SARS-CoV-2 PCR and anti-SARS-CoV-2 IgG negative. Additional virological and microbiological diagnostics negative. NMDAR- and GAD-65- antibodies positive (immunoblotting revealed IgG against NMDA receptor and GAD65/67 -> cell-based indirect immunofluorescence assay revealed cell-surface and intracellular antibody binding).
	EEG	Subcortical dysfunction in frontal, temporal and occipital regions
	Brain imaging	MRI: T2 FLAIR and diffusion-weighted imaging: bilateral hyperintensities in the anterior cingulated cortex and temporal lobes
	Therapy	Anti-seizure treatment After NMDAR encephalitis diagnosis, methylprednisolone and intravenous immunoglobulin
	Course	Physical rehabilitation, presents neurological sequelae related to mood disorders, irritability and agitation
Patient 3	Khoreva et al. [70]	Female (16 years old)
	Reason for hospitalisation	Fever
	Respiratory (other) symptoms	COVID-19 positive (SARS-CoV-2 PCR positive), mild symptoms, fever, headache, nausea, tachycardia and tachypnoea. Ovarian teratoma
	Neuropsychiatric symptoms	Personality changes, aggression, visual hallucinations, agitation, memory impairment, catatonia, epileptic seizure, coma
	Blood test	Not mentioned
	CSF examination	Lymphocytic pleocytosis (62 cells/µl), normal white cell count in second lumbar puncture, normal protein. No SARS-CoV-2 PCR testing performed. Additional virological and microbiological diagnostics negative, IgG-NMDAR-antibodies positive (cell-based indirect immunofluorescence assay)
	EEG	Not explicitely mentioned, likely abnormal due to multiple seizures.
	Brain imaging	MRI: normal (contract agent was used); FDG-PET: hyper-metabolism in the basal nuclei and cerebellum, diffuse cortical hypometabolism.
	Therapy	Antiviral, antibacterial, antipsychotic and antiseizure therapy Methylprednisolone and intravenous immunoglobulin Removal of teratoma
	Course	Antiseizure therapy and rehabilitation
Patient 4	Bazalar et al. [71]	Female (19 years old)
	Reason for hospitalisation	Psychiatric symptoms
	Respiratory (other) symptoms	COVID-19 positive (SARS-CoV-2 PCR positive), mild flu symptoms. Examination for ovarian teratoma not mentioned.
	Neuropsychiatric symptoms	Personality changes, aggression, audio-visual hallucinations, agitation, catatonia, epileptic seizure, coma
	Blood test	Leucocytosis and elevated CRP
	CSF examination	White celll count 95/µl, 70% lymphocytes. SARS-CoV-2 not mentioned. Additional virological and microbiological diagnostics negative. IgG-NMDAR-antibodies positive (cell-based indirect immunofluorescence assay).
	EEG	Diffuse, slow activity, intermittent delta waves
	Brain imaging	MRI: normal (no contrast agent was used)
	Therapy	Antipsychotic treatment Antiviral prophylactic and antiseizure therapy Methylprednisolone, intravenous immunoglobulin and rituximab
	Course	marked improvement, after sixth dose of rituximab, discharged
Patient 5	Gillentine et al. [73]	Female (37 years-old)
	Reason for hospitalization	Headache, general malaise, fever and upper respiratory tract symptoms COVID-19-positive (test method not mentioned) 6 weeks earlier with uncomplicated course
	Respiratory (other) symptoms	Severe respiratory distress, subsequently intubated Benign ovarian teratoma of left ovary
	Neuropsychiatric symptoms	Visual hallucinations and paranoid behaviour, increased agitation, catatonia
	Blood test	Leucocytosis
	CSF examination	Lymphocytic pleocytosis (white cell count not shown) and elevated protein. SARS-COV-2 antibodies negative. Additional virological and microbiological diagnostics negative. IgG NMDAR-antibodies positive (cell-based indirect immunofluorescence assay).
	EEG	Epileptic focus in the right parietal region
	Brain imaging	MRI: normal (unclear if contrast agent was used)
	Therapy	Antiviral and antibacterial treatment Later steroids, intravenous immunoglobulins and rituximab Prophylactic bilateral salpingo-oophorectomy
	Course	After 56 hospital days the patient died
Patient 6	Kaur et al. [80]	Male (10 months old)
	Reason for hospitalization	Poor feeding and irritability for 5 days, 2 episodes of convulsion
	Respiratory (other) symptoms	Moderate symptoms: 40 days before admission upper respiratory tract infection, SARS-COV-2 IgG strongly positive
	Neuropsychiatric symptoms	Poor interactions with caregivers Extrapyramidal movements with generalized and oro –linguo-buccal dystonia with athetosis
	Blood test	Leucocytosis, SARS-COV-2 IgG strongly positive
	CSF examination	White cell count 20/µl, 95% lymphocytes. SARS-CoV-2 not mentioned. Additional virological and microbiological diagnostics negative. NMDAR-antibodies positive (Ig class unclear, cell-based indirect immunofluorescence assay).
	EEG	Not mentioned
	Brain imaging	MRI: normal (unclear if contrast agent was used)
	Therapy	Methylprednisolone, intravenous immunoglobulin and rituximab Azathioprine, cyclophosphamide and extrapyramidal movements treatment
	Course	Extrapyramidal movements well controlled and baby regained age-appropriate milestones
Patient 7	Alvi et al. [74]	Female (13 years old)
	Reason for hospitalization	Behavioural issues for one week with intermittent aggression and irritability with disturbed sleep/wake cycle No CoViD-19 vaccination
	Respiratory (other) symptoms	No respiratory or gastrointestinal symptoms, tachycardiac MRI of pelvis and abdomen were unremarkable
	Neuropsychiatric symptoms	Three episodes of generalized tonic clonic seizures with worsened conscious level
	Blood test	SARS-COV-2 antibodies (type of antibodies not mentioned) positive
	CSF examination	Normal protein and cell quantity. SARS-CoV-2 not mentioned. Additional virological and microbiological diagnostics negative. NMDAR-antibodies positive (Ig class and test method not mentioned).
	EEG	Delta brush pattern
	Brain imaging	MRI: normal (unclear if contrast agent was used)
	Therapy	Anti-seizure treatment Methylprednisolone, plasmapheresis and rituximab
	Course	Residual deficit in executive function still present
Patient 8	Naidu and Tayler [75]	Female (50 years old)
	Reason for hospitalization	Fever, cough, myalgia
	Respiratory (other) symptoms	COVID-19 positive (test method not mentioned). No respiratory symptoms. Severe nausea and vomiting Ovarian tumors were excluded
	Neuropsychiatric symptoms	Progressive weakness of all limbs, confusion and disorganised behaviour Later emotional lability, quadriparesis, left sided sensory deficits, slurred speech, urinary retention and cognitive impairment
	Blood test	Elevated CRP, increased D-dimer
	CSF examination	White cell count 0/µL (no pleocytosis), elevated protein and IgG. SARS-CoV-2 not mentioned. Additional virological and microbiological diagnostics negative. IgG NMDAR-antibodies positive (cell-based indirect immunofluorescence assay).
	EEG	Abnormal EEG: bilateral fronto-central delta to theta slowing noted intermittently. No delta brushes seen. Intermittent right sided P4 epileptiform sharp wave activity with field spread.
	Brain imaging	MRI with gadolinium: bilateral hyper-intense T2 and FLAIR lesions in the corticospinal tracts
	Therapy	Valproate, corticosteroids, intravenous immunoglobulin and plasmapheresis Rituximab as long term therapy
	Course	Significant improvement of symptoms.
Patient 9	Khubetova [81]	Male (45 years old). Chronic kidney failure Dialysis
	Reason for hospitalization	Severe lethargy, speech and swallowing disorders
	Respiratory (other) symptoms	No respiratory symptoms. COVID-19 positive (test method not mentioned), creatinine increase
	Neuropsychiatric symptoms	Bradyphrenia, bradylalia, bradykinesia, hyposmia, bilateral achyrokinesis
	Blood test	Elevated CRP and IL-6. NMDAR-antibodies positive (Ig class and test method not mentioned).
	CSF examination	Unclear if NMDAR-antibodies were tested in CSF or only in blood (see above).
	EEG	Not mentioned
	Brain imaging	MRI: multiple foci of gliosis in both hemispheres, probably of vascular origin (unclear if contrast agent was used)
	Therapy	Amantadine, citicoline
	Course	Improvement of neurological symptoms
Patient 10	Lee et al. [68]	Female (20 years old)
	Reason for hospitalisation	Convulsive seizure
	Respiratory (other) symptoms	No symptoms, COVID-19 negative 3 days before admission: vaccination with BNT162b2, expression of SARS-CoV-2 spike protein in cells by vaccination with mRNA (Pfizer-BioNTech) Bilateral ovarian teratoma
	Neuropsychiatric symptoms	Repetitively asking the same questions, Sent inappropriate text messages to friends Disturbances of comprehension and reading, seizure.
	Blood test	NMDAR-antibodies positive (test method or type of antibodies not mentioned)
	CSF examination	White cell count 58/µl, mainly lymphocytic, mild elevation of protein, Virological diagnostics negative. IgG NMDAR-antibodies positive (cell-based indirect immunofluorescence assay).
	EEG	Frequent spike-and-waves in bilateral temporal areas
	Brain imaging	MRI: normal except leptomeningeal enhancement and gyral swelling in both cerebral convexity on T1-weighted contrast-enhanced images and subtle signal change on T2-weighted and FLAIR images
	Therapy	Antiviral prophylactic therapy After NMDAR encephalitis diagnosis, methylprednisolone, intravenous immunoglobulin and rituximab Removal of teratoma
	Course	Fully recovered
Patient 11	Flannery et al. [69]	Female (in her 20’s)
	Reason for hospitalisation	Urinary frequency (subjective reason) and anxiety
	Respiratory (other) symptoms	No symptoms, COVID-19 negative 1 week before admission: vaccination with BNT162b2, expression of SARS-CoV-2 spike protein in cells by vaccination with mRNA (Pfizer-BioNTech) Chest x-ray and CT, and MRI of the chest, pelvis (exclusion of ovarian teratoma), and abdomen were unremarkable During admission, tachycardia and hypertension
	Neuropsychiatric symptoms	Anxiety, decreased mentally acuity, insomnia and fixation that she suffered from irritable bowel and kidney disease Motor dysfunction and transient aphasia Later auditory hallucinations, psychotic symptoms and catatonia
	Blood test	Mild leucocytosis, increased alanine aminotransferase and aspartate aminotransferase. NMDAR-antibodies positive (commercial IgG assay)
	CSF examination	White cell count 7/µL, 89% lymphocytes. Virological diagnostics negative. IgG NMDAR-antibodies positive (cell-based indirect immunofluorescence assay).
	EEG	No abnormalities
	Brain imaging	MRI: normal (unclear if contrast agent was used)
	Therapy	Antipsychotic treatment Methylprednisolone, intravenous immunoglobulin and rituximab Metoprolol to manage tachycardia and hypertension
	Course	Absence of seizures, improvement of neurological symptoms
Patient 12	Hina Naz et al. [72]	Female (33 years old)
	Reason for hospitalisation	Psychiatric symptoms
	Respiratory (other) symptoms	COVID-19 negative 1 day before admission: vaccination with BBIBP-CorV, whole inactivated virus (Sinopharm). Mild flu-like symptoms. Pelvis MRI was unremarkable
	Neuropsychiatric symptoms	Personality changes, memory impairment, aggression, audio-visual hallucinations, agitation, catatonia, epileptic seizure, coma
	Blood test	Polyclonal IgG bands
	CSF examination	White cell count 363/µl, 94% lymphocytes, elevated protein. Oligoclonal IgG bands positive in CSF. Virological and microbiological diagnostics negative. NMDAR-antibodies positive (Ig class and test method not mentioned).
	EEG	Diffuse, slow activity, bilateral spikes and slow waves discharges in frontocentral regions
	Brain imaging	MRI: normal (contract agent was used)
	Therapy	Antiviral prophylactic and antiseizure therapy Methylprednisolone, intravenous immunoglobulin and rituximab
	Course	Fully recovered
Patient 13 (Patient 1 in [45])	Panariello et al. [82]	Male (23 years-old). History of drug abuse
	Reason for hospitalisation	Psychomotor agitation, anxiety, formal thought disorder, persecutory delusions and auditory hallucinations and global insomnia. SARS-CoV-2 positive nasopharyngeal swab RT-PCR testing
	Respiratory (other) symptoms	Moderate fever, drop in O_2_ saturation, chest X-ray: bilateral milk glass opacities, chest CT: patchy bibasilar consolidation
	Neuropsychiatric symptoms	Confusion, disorganization of speech, thought/behaviour, auditory hallucinations and insomnia. Week 2: mutistic/non-responsive. Week-3: dysphagia, dyskinesia, autonomic instability, fluctuations in body temperature, blood pressure, pulse and respiratory rate
	Blood test	CRP elevated, leukocytosis, hyponatremia at time of NMDAR encephalitis diagnosis
	CSF examination	Red and white cell count elevated (960/µL, artificial?), elevated protein. SARS-CoV-2 negative (PCR test). Additional virological and microbiological diagnostics negative. IL-6 elevated, IgG-NMDAR-antibodies positive (cell-based indirect immunofluorescence assay).
	EEG	Theta activity, unstable, non-reactive to visual stimuli
	Brain imaging	CT scan was negative for neuroanatomical acute abnormalities.
	Therapy	Seizure prophylaxis. No symptom improvement with antipsychotics. COVID-19 therapy with hydroxychloroquine and darunavir/cobicistat. Antibiotic prophylactic therapy. After NMDAR encephalitis diagnosis, dexamethasone and intravenous immunoglobulin
	Course	Clinical symptoms improved
Patient 14 (Patient 2 in [45])	Alvarez Bravo and Ramio [76]	Female (30 years-old). No previous medical history. SARS-CoV-2 positive nasopharyngeal swab RT-PCR testing
	Reason for hospitalisation	Behavioral changes
	Respiratory (other) symptoms	Severe fever, pneumonia, thrombosis of the left iliac vein, and bilateral pulmonary embolism attributed to SARS-CoV-2 infection. Ovarian teratoma
	Neuropsychiatric symptoms	Psychomotor agitation, paranoid ideation, dysarthria with dysprosody, and visual hallucinations, focal and generalised seizures
	Blood test	Routine blood test: normal.
	CSF examination	White cell count 44/µL, 90% lymphocytes, protein elevated. SARS-CoV-2 negative (PCR test), Additional virological and microbiological diagnostics negative. IgG NMDAR antibodies positive (cell-based indirect immunofluorescence assay).
	EEG	Delta brush pattern, epileptic discharges in the left frontotemporal region
	Brain imaging	Hyperintensities in the left hippocampus in T2-weighted FLAIR sequences
	Therapy	After NMDAR encephalitis diagnosis, 5 days of methylprednisolone and immunoglobulins administered. Ovarian teratoma was removed by laparotomy.
	Course	Hypoprosexia, emotional lability and memory disorder, Stabilised systemic and respiratory symptoms
Patient 15 (Patient 3 in [45])	Allahyari et al. [77]	Female (18 years-old). No previous medical history. SARS-CoV-2 positive nasopharyngeal swab RT-PCR testing
	Reason for hospitalisation	Generalized tonic-clonic seizures
	Respiratory (other) symptoms	Moderate fever, pneumonia, hypotonia, tachycardia, tachypnea, oxygen saturation of 90%, bilateral pulmonary crackles in lower lung zones, Examination for ovarian teratoma not mentioned.
	Neuropsychiatric symptoms	3-week history of mood change as depression and anhedonia accompanied by lack of concentration, generalized tonic–clonic seizures
	Blood test	Neutrophilia, lymphopenia, CRP elevated
	CSF examination	White cell count 27/µL, 93% lymphocytes. SARS-CoV-2 positive (PCR test). Additional virological and microbiological diagnostics negative. IgG NMDAR antibodies positive (cell-based indirect immunofluorescence assay).
	EEG	Epileptic discharges in the left frontotemporal region
	Brain imaging	MRI: normal
	Therapy	Seizure prophylaxis. COVID-19 therapy with Remdesivir, Lopinavir/Ritonavir, and Interferon b1a (Resi- gen). Antibiotic prophylactic therapy. After NMDAR encephalitis diagnosis, methylprednisolone and intravenous immunoglobulin
	Course	After 2 months of hospitalization discharged with full recovery
Patient 16 (Patient 5 in [45])	Monti et al. [83]	Male (50 years-old). Moderate arterial hypertension
	Reason for hospitalisation	Acute psychiatric symptoms. SARS-CoV-2 positive nasopharyngeal swab RT-PCR testing
	Respiratory (other) symptoms	No respiratory symptoms. No diarrhoea. Fever present
	Neuropsychiatric symptoms	Confabulations and delirium. Day-4: focal motor seizures with reduced consciousness, orofacial dyskinesia, automatisms. Sudden refractory status epilepticus
	Blood test	IL-6 elevated. No CRP elevation or leukocytosis
	CSF examination	Three lumbar punctures: White cell count 76/µL and 25/µL, oligoclonal bands. SARS-CoV-2 not mentioned. Additional virological and microbiological diagnostics negative. Third lumbar puncture: IgG NMDAR antibodies positive (cell-based indirect immunofluorescence assay), IL-6 elevated.
	EEG	Diffuse delta activity with extreme delta brush pattern. Anterior subcontinuous periodic theta activity
	Brain imaging	MRI: normal
	Therapy	Antiepileptics and anaesthetics. COVID-19 therapy with hydroxychloroquine and lopinavir/ritonavir. After diagnosis of NMDAR encephalitis: corticosteroids, immunoglobulins and plasmapheresis
	Course	4 months after symptom onset patient discharged in good condition with no neuropsychiatric symptoms
Patient 17 (Patient 6 in [45])	Burr et al. [78]	Female (23 months-old). Vaccinated. No previous diseases. Family history unremarkable
	Reason for hospitalisation	Fever, psychomotor agitation, was no longer talking, sleep disturbances, constipation, decreased oral intake. SARS-CoV-2 positive nasopharyngeal swab RT-PCR testing
	Respiratory (other) symptoms	No respiratory symptoms. Fever, dehydration present. Examination for ovarian teratoma not mentioned.
	Neuropsychiatric symptoms	Agitation, poor sleep, mood swings, mutism, regular kicking/ fapping of extremities. Day-2: multiple epileptic seizures. Week 2: worsening encephalopathy with persistent hyperkinetic movements of extremities and head
	Blood test	CRP normal, NMDAR antibodies (IgG) positive (test method not mentioned).
	CSF examination	White cell count 7/µL, 89% lymphocytes, oligoclonal bands not tested. SARS-CoV-2 negative (PCR test). Additional virological and microbiological diagnostics negative. IgG NMDAR antibodies positive (cell-based indirect immunofluorescence assay).
	EEG	Not mentioned
	Brain imaging	MRI with and without contrast: normal
	Therapy	Antiepileptics. After NMDAR encephalitis diagnosis, corticosteroid therapy for 5 days with no improvement, followed by intravenous immunoglobulin administration
	Course	Remission within one week after immunoglobulin therapy
Patient 18 (Patient 7 in [45])	Sanchez-Morales et al. [84]	Male (14 years-old). No previous medical history. SARS-CoV-2 positive nasopharyngeal swab RT-PCR testing
	Reason for hospitalisation	Behavioural changes and neurological symptoms
	Respiratory (other) symptoms	None
	Neuropsychiatric symptoms	Altered behaviour and mental status, epileptic seizures, insomnia, orolingual dyskinesia
	Blood test	SARS-CoV-2 IgM and IgG negative
	CSF examination	White cell count 2/µL (no pleocytosis). SARS-CoV-2 positive (PCR test and IgG). Additional virological and microbiological diagnostics negative. NMDAR antibodies positive (Ig class and test method not mentioned).
	EEG	Not mentioned
	Brain imaging	MRI was done, findings were not mentioned
	Therapy	After NMDAR encephalitis diagnosis, methylprednisolone and immunoglobulins administered
	Course	Complete remission of neurological impairment. Control of epilepsy. Persistence of psychiatric symptoms
Patient 19 (Patient 8 in [45])	Sarigecili et al. [85]	Male (7 years-old). Vaccinated. No previous diseases. No abnormal family history
	Reason for hospitalisation	Gait disorder. SARS-CoV-2 positive nasopharyngeal swab RT-PCR testing
	Respiratory (other) symptoms	None. No headache, fever, or cold symptoms. Day 8: tachycardia
	Neuropsychiatric symptoms	Ataxia and broad-based gait with poor muscle reflexes. Day-2: somnolence and epileptic seizures. Day 8: choreiform movements of extremities, tongue protrusion, bruxism, smacking, psychomotor agitation, catatonia, echolalia
	Blood test	CRP elevated, lymphopenia. IL-6 not mentioned
	CSF examination	No cells present, oligoclonal bands negative. SARS-CoV-2 not mentioned. Additional virological and microbiological diagnostics negative. IgG NMDAR antibodies positive (test method not mentioned). IL-6 not mentioned
	EEG	Encephalopathic pattern with disseminated delta waves
	Brain imaging	MRI: normal
	Therapy	Antiepileptics after onset of seizures. Initial therapy with antibiotics/antivirals. After diagnosis of NMDAR encephalitis: plasmapheresis three times, corticosteroid 7 days, immunoglobulins 5 days followed by corticosteroid again
	Course	Day 31: patient discharged walking but mildly ataxic with prednisolone and antiepileptic treatment. Possibility of repeat immunoglobulin administration

### Demographics

Of the 19 cases, 12 were females ranging from 23 months to 50 years-old [67–78] and 7 were males ranging from 10 months to 50 years-old [79–85] (Table 1). Two articles were in Spanish [71, 76], one was in Russian [70], one in Ukrainian [81], and the rest were published in the English language. Sixteen of the 19 cases tested positive for COVID-19 either before or shortly after admission (Table 1), and three patients had received a COVID-19 vaccination <1 week before admission [68, 69, 72].

### Reason for hospitalization

All patients described in the published case reports had psychiatric and/or neurological symptoms and had been hospitalized for various reasons. These included: seizures [67, 68, 77]; front-orbital syndrome characterized by catatonic symptoms [79]; fever [70, 73, 75, 78]; psychiatric symptoms [71, 72, 76, 78, 82–84]; urinary frequency [69]; poor feeding, irritability and convulsion [80]; disturbed sleep [74, 78, 82]; gait disorder [85]; and severe lethargy, speech and swallowing difficulty [81]. Six out of the 16 patients with COVID-19 positivity had no respiratory symptoms [74, 75, 78, 81, 83, 84], while 10 of them had mild, to severe respiratory symptoms (Table 1).

### CSF and blood findings

#### CSF

All cases were positive for the presence of NMDA receptor antibodies in CSF (Table 1), while positive test results for antibodies in both CSF and blood samples were mentioned in two cases [68, 78]. In addition, two patients showed oligoclonal immunoglobulin G (IgG) bands in CSF [73, 83] and one of these had polyclonal IgG banding in blood [72]. Another patient was also positive for the presence of glutamate decarboxylase 65 kDa isoform (GAD-65) antibodies in CSF [79]. In addition, pleocytosis >5/μL was found in 10 patients [69, 70, 72, 73, 76–78, 80, 82, 83]. Four cases had high protein and pleocytosis [69, 73, 76, 79] and one patient had only high protein levels in their CSF [75]. All cases were negative for commonly used virological and microbiological tests in CSF (excluding SARS-CoV-2 testing).

#### Blood

Some cases also showed elevations in other blood measures. For example, four cases had leukocytosis [71, 73, 80, 82] and two had lymphopenia [77, 85]. Other cases showed elevations in liver aminotransferases [69]; C-reactive protein (CRP) [77, 82, 85]; and one had high creatinine [81].

### EEG recordings

EEG abnormalities occurred in 13 patients and consisted of either frontal intermittent rhythmic delta activity (FIRDA) [67], spike-and-waves in bilateral temporal areas [68], subcortical dysfunction in frontal, temporal and occipital regions [79], diffuse, slow activity, intermittent delta waves [71], delta brush pattern [74], diffuse delta activity with extreme delta brush pattern and anterior subcontinuous periodic theta activity [83], encephalopathic pattern with disseminated delta waves [85], diffuse, slow activity, bilateral spikes and slow waves discharges in fronto-central regions [72], epileptic focus in the right parietal region [73], epileptic discharges in the left frontotemporal region [76, 77], theta activity, unstable and non-reactive to visual stimuli [82]. The remaining cases were either normal for EEG recordings [69], this analysis was not mentioned or performed, or the analysis was not mentioned but likely abnormal due to the presence of multiple seizures [70].

### Imaging

#### Brain imaging

Brain imaging was performed all cases described. Eighteen cases had MRI and one had a computed tomography (CT) scan of the brain. Unfortunately, it was often not clear whether an examination with a contrast agent had also been carried out. Twelve MRI scans were classified as normal [67, 69–74, 77, 78, 80, 83, 85], and one patient presented normal findings except for some subtle signal changes on T2-weighted and FLAIR images [68]. Another case showed hypermetabolism in the basal nuclei and diffuse cortical hypometabolism in FDG-PET [70]. One patient had changes in brain morphology due to multiple gliosis of vascular origin [81], and two patients presented typical NMDA receptor encephalitis findings and hyperintensities in T2 and/or FLAIR sequences [76, 79]. The patient who had the CT scan was classified as normal [82].

#### Presence of ovarian teratoma

In the 12 female patients, four cases screened for ovarian teratoma showed the presence of this malignancy [68, 70, 73, 76]. However, 5 other cases were examined for the presence of ovarian teratomas and were negative [67, 69, 72, 74, 75]. Three publications did not mention a search for ovarian teratoma [71, 77, 78].

### Outcome

Seventeen out of the 19 cases received intravenous immunoglobulin (IVIg) and corticosteroids after the NMDA receptor encephalitis diagnosis [67–73, 75–80, 82–85]. In addition, four cases also received plasmapheresis therapy [74, 75, 83, 85], 9 were administered rituximab [67–69, 71–75, 80], 12 cases were recorded as having received anti-seizure treatment [67, 70–72, 74, 75, 77–79, 82, 83, 85], and 5 were given antipsychotics [67, 69–71, 82]. One patient received corticosteroids, plasmapheresis and rituximab [74] and one was treated with amantadine and citicoline [81]. Nine patients were given antiviral medication [68, 70–73, 77, 82, 83, 85] and four had an ovarian teratoma removed [68, 70, 73, 76]. Finally, one patient was given metoprolol to manage tachycardia and hypertension [69], and one received azathioprine, cyclophosphamide and treatment for extrapyramidal movements [80].

Of the 19 cases, patient outcome was not mentioned in one study [67], two patients showed no improvement [74, 79], 15 improved or fully recovered [68–72, 75–78, 80–85] and one died [73].

## Proposed mechanisms of NMDA receptor encephalitis following SARS-CoV-2

While general effects of SARS-CoV-2 on neuroinflammation are evident through peripheral inflammation and blood-brain barrier (BBB) disruption, autoimmune CNS effects of the virus may also play a role, although these are less well understood. Further research in both areas is essential for a more comprehensive understanding.

### SARS-CoV-2, peripheral inflammation and BBB permeability

SARS-CoV-2 primarily targets respiratory epithelial cells but can stimulate a broad inflammatory response. Once the virus has been detected by the immune system, a cascade of inflammatory cytokines and chemokines, sometimes termed a “cytokine storm,” is released, which can instigate systemic inflammation [86]. Inflammatory mediators from the periphery can compromise the BBB, e.g. through the effects of pro-inflammatory cytokines or activation of the kynurenine pathway [87, 88]. Alternatively, direct effects of SARS-CoV-2 on the BBB have been proposed [89]. Finally, the breakdown of the BBB can trigger neuroinflammation, including the recruitment of macrophages and lymphocytes and the activation of astrocytes and microglia [88].

### Induction of CNS autoimmunity by SARS-CoV-2

The precise mechanism of how SARS-CoV-2 viral infection can lead to autoimmunity in the CNS is not known. In cases of NMDA receptor encephalitis, it is possible that viral proteins such as NSP8 and NSP9 released during the infection cycle may lead to molecular mimicry of host interactions of the NR2A and NR1 subunits of the NMDA receptor [45]. In turn, this may lead to bystander T cell activation and epitope spreading, giving rise to an autoimmune reaction, as proposed by Pacheco et al. [90]. As an outcome of this, the virus-associated immune response in vascular endothelia can cause disruption of the BBB, permitting entry of these antibodies into the CNS in rare cases. This can lead to further BBB disruption and inflammation, along with a cellular immune response in the brain. Finally, auto-antibodies originating from the periphery or produced in the CNS can cross-react with NMDA receptor subunits leading to down regulation of this neuronal circuity.

Psychosis resulting from the presence of NMDA receptor antibodies is thought to result from (i) increased opening of synaptic NMDA receptor channels, and (ii) subsequent movement of these to extra-synaptic locations or via internalization [91]. As these channels open less frequently, this can lead to an imbalance between synaptic and extra-synaptic NMDA receptors. This imbalance is proposed to result in the multifaceted neurological presentations of NMDA receptor encephalitis [91]. A comparable effect can also be observed with administration of ketamine or phencyclidine as well as by antibodies against the NR1 and NR2 NMDA receptor subunits [92].

## Diagnosis and treatment

To guide clinical decision making, step-wise schemes have been developed which can facilitate timely and accurate diagnostics to allow early initiation of the appropriate therapeutics for autoimmune encephalitis patients [48, 93–95]. If the patient presents with new-onset progressive psychiatric symptoms, together with focal neurological abnormalities, testing for the presence of autoantibodies should be considered depending on the results from the basic CSF analyses, in addition to EEG and MRI if warranted. To support this work, several multiplex immunoassay screening panels are available which can test for the presence of autoantibodies against NMDA, α-amino-3-hydroxy-5-methyl-4-isoxazolepropionic acid (AMPA) and gamma-aminobutyric acid (GABAB) receptors (EUROIMMUN; Lübeck, Germany [96]) as well as the M1, M2 and M5 muscarinic acetylcholine, and α1- and α2- adrenergic receptors (CellTrend; Berlin, Germany [97]).

**Fig. 2:**
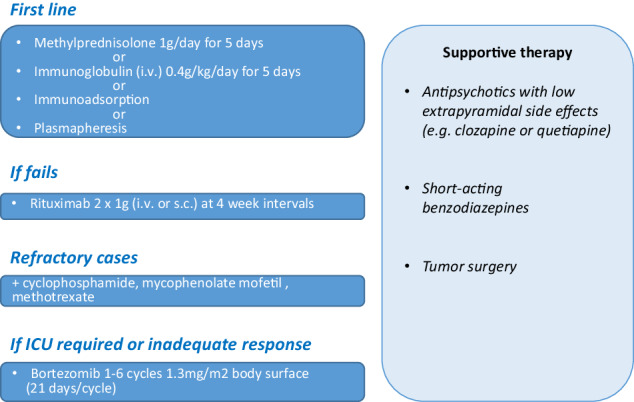
Treatment guide for NMDA receptor encephalitis following SARS-CoV-2 infection considering international expert recommendations. Immunosuppression by corticosteroid therapy (1 g methylprednisolone/day for 5 days), intravenous human immunoglobulin (IVIg) administration (0.4 g/kg/day for 5 days) or immunoadsorption or plasmapheresis for rapid removal of pathogenic autoantibodies should be first-line therapy in patients with definite autoimmune encephalitis. If therapy fails, treatment should be extended within a few days, preferably with rituximab (2 × 1000 mg i.v. or s.c. at 2 to 4-week intervals). In refractory cases, combination treatment with cyclophosphamide (750 mg/m^2^ body surface area every 4 weeks), mycophenolate mofetil, or methotrexate may also be required to achieve a clinical response. Bortezomib can be used for escalation therapy (1–6 cycles of 1.3 mg/m^2^ body surface area for 21 days each cycle). In addition, antipsychotics with low extrapyramidal side effects (quetiapine, clozapine) should be prescribed for symptomatic pharmacotherapy of psychotic symptoms to reduce the risk of neuroleptic-induced dyskinesia or malignant neuroleptic syndrome. Short-acting benzodiazepines can be used for anxiolysis, sedation and to treat catatonic symptoms [91, 94, 98].

The standard first treatment for autoimmune encephalitis is high-dose steroids over a 3 to 5-day course, sometimes in combination with administration of intravenous immunoglobulins or plasmapheresis (Fig. 2). In case of the need for second line or escalation therapies, treatment with rituximab can be employed [94, 98]. In refractory cases, immunosuppressive agents such as cyclophosphamide, mycophenolate mofetil or methotrexate can be used to achieve an effective response [94, 98]. If there is no response to above line of therapy, treatment with the proteasome inhibitor and immunosuppressant bortezomib can be attempted [99].

## Critical considerations regarding relevance of SARS-CoV-2-induced molecular mimicry of NMDA receptors and questions of causality

At present, there are still open questions regarding classification of the clinical relevance of SARS-CoV-2-induced molecular mimicry of NMDA receptors and whether there is a causal relationship between SARS-CoV-2 infection or vaccination and the manifestation of NMDA receptor encephalitis. Based on the current literature this should be considered very rare, consistent with our finding of only 19 case reports worldwide during the COVID-19 pandemic.

The first question is whether antineuronal NMDA receptor antibodies are found more frequently in blood after acute COVID-19 disease or SARS-CoV-2 vaccination compared to other infections with similar severity. For example, these antibodies were present in blood more frequently if there was a serum scar from previous influenza infections [100]. However, a recent study of thousands of serum samples found no change in the positivity rate of NMDA receptor antibodies during the COVID 19 pandemic [101].

Notably, the presence of NMDA receptor antibodies in serum alone does not cause encephalitis, as CNS-reactive antibodies must be present in brain tissue to cause CNS symptoms. Approximately 10% of people without neuropsychiatric disease have low titers of NMDA receptor serum antibodies. This is age dependent as >20% of older individuals can show the presence of these antibodies, probably because their immune system matured further by contact with an increasing number of pathogens or antigens in general in the course of life [100]. This leads to the second question of whether encephalitis occurs more frequently after acute COVID-19 disease or SARS-CoV-2 vaccination compared to other infections with similar severity. For this to happen, the antibodies either reach the brain or are produced intrathecally [102].

Given that more than half of the world’s population had COVID-19 and more than 12.7 billion vaccinations have been performed [103], the publication of 19 case reports worldwide with a possible NMDA receptor antibody-mediated CNS disease suggests that this is exceedingly rare. Thus, the specific association between COVID-19 and NMDA receptor encephalitis is weak.

An important confounding factor is that ovarian teratoma was identified as a comorbidity in four of the 12 women. This tumor is a known trigger for development of NMDAR encephalitis [98]. Six other cases were examined and had no ovarian teratomas, while no search for this tumor was mentioned in two publications. As the current reports are not complete regarding screening for ovarian teratomas or potential pre-existence of NMDA receptor autoantibodies, it is not clear if a causal relationship existed between SARS-CoV-2 infection or vaccination and the NMDA receptor encephalitis diagnosis. Also, in the patient with focal right frontal lobe epilepsy [67], it is not clear if the NMDA receptor antibody formation was triggered by tissue destruction in the context of stroke or epileptic seizures^1^ rather than by SARS-CoV-2 infection. As far as vaccination-related causality is concerned, it should be noted that the interval between vaccination and hospitalisation was short (1 to 7 days). This raises the question of whether a transfer of pre-formed serum antibodies into the brain or CSF through BBB opening by pro-inflammatory cytokines during the immune response to vaccines played a role, rather than de novo NMDA receptor antibody formation induced by vaccination [102]. Notably, one of the cases with vaccination also had comorbid ovarian teratoma [68].

Nationwide studies including all SARS-CoV-2 PCR tests performed in Denmark showed that the risk of new-onset psychiatric and neurological disorders increased after COVID-19, particularly in association with disease severity [104, 105]. However, the risk was comparable to that observed after other infections of similar severity [104, 105]. An unbiased epidemiological analysis of the manifestation of NMDA receptor encephalitis after COVID-19 or vaccination is still lacking but this would be important for clarification regarding a causal link. Unfortunately, there is no specific ICD-10 code used only for autoimmune encephalitis or specifically for NMDA encephalitis, which makes it more difficult to extract such numbers from registers. Thus, there is a need to go into large-scale electronic health records to further shed light on the associations on a large-scale. However, in studies examining patients with severe COVID-19 and neuropsychiatric symptoms, NMDA receptor antibodies did not consistently appear in the CSF, so they are considered rare [106]. Also, CNS infection with SARS-CoV-2 have only been described on a case report level, whereas the CSF screening studies have not found clear evidence of intrathecal SARS-CoV-2 antibody production since levels in the peripheral blood were higher [106].

On the other hand, a Barcelona-based research network on autoimmune encephalitis found that the annualized mean number of patients screened for any type of antineuronal antibodies increased 1.3 times during the COVID-19 pandemic compared to the pre-pandemic period (*p* < 0.001) [107]. The overall positivity rate, controlled for the number of patients examined per year, did not change significantly during the COVID-19 pandemic (*p* = 0.51), but sub-analyses for different antibody specificities showed a potential increase in NMDA receptor positive serum and CSF findings (*p* = 0.03).

Important for assessing the incidence of autoimmune encephalitis after COVID-19 vaccination is the insight provided by large databases, such as the publicly available EudraVigilance database of the European Medicines Agency [108].^2^ This database contains detailed safety information on all medicines approved in Europe, such as the COVID-19 vaccines. Although NMDA receptor encephalitis is not specifically listed, a search for reports of “autoimmune encephalitis” associated with COVID-19 vaccines on 28 August 2023 revealed the following numbers [108, 109]:

- COVID-19 mRNA vaccine ORIGINAL (Elasomeran; Moderna): ~55 million doses were distributed in Europe. Overall ~380.000 reports of suspected side effects; 23 reports of suspected autoimmune encephalitis associated with this vaccine by healthcare professionals.
- COVID-19 mRNA vaccine ORIGINAL/OMICRON BA.1 (Elasomeran/Imelasomeran; Moderna): ~12 million doses were distributed in Europe. Overall 675 reports of suspected side effects; 0 reports of suspected autoimmune encephalitis associated with this vaccine.
- COVID-19 mRNA vaccine ORIGINAL (Tozinameran; Pfizer-Biontech): ~137 million doses were distributed in Europe. Overall ~1.2 Million reports of suspected side effects; 62 reports of suspected autoimmune encephalitis associated with this vaccine by healthcare professionals.
- COVID-19 mRNA vaccine ORIGINAL/OMICRON BA.1 (Tozinameran/ Riltozinameran; Pfizer-Biontech): ~9.5 million doses were distributed in Europe. Overall ~6.000 reports of suspected side effects; 0 reports of suspected autoimmune encephalitis associated with this vaccine.
- COVID-19 mRNA vaccine ORIGINAL/OMICRON BA.4-5 (Tozinameran/ Famtozinameran; Pfizer-Biontech): ~23.5 million doses were distributed in Europe. Overall ~9.400 reports of suspected side effects; 2 reports of suspected autoimmune encephalitis associated with this vaccine by healthcare professionals.
- COVID-19 vector vaccine CHADOX1 NCOV-19 (AstraZeneca): ~12 million doses were distributed in Europe. Overall ~550.000 reports of suspected side effects; 28 reports of suspected autoimmune encephalitis associated with this vaccine by healthcare professionals.
- COVID-19 vector vaccine AD26.COV2.S (Janssen): ~17.5 million doses were distributed in Europe. Overall ~71.000 reports of suspected side effects; 3 reports of suspected autoimmune encephalitis associated with this vaccine by healthcare professionals.
- COVID-19 recombinant spike protein vaccine NVX-COV2373 (Novavax): ~3.2 million doses were distributed in Europe. Overall ~1.600 reports of suspected side effects; 0 reports of suspected autoimmune encephalitis associated with this vaccine.
- COVID-19 inactivated virus vaccine VLA2001 (Valneva): ~150.000 doses were distributed in Europe. Overall 34 reports of suspected side effects; 0 reports of suspected autoimmune encephalitis associated with this vaccine.
- COVID-19 recombinant spike protein vaccine VIDPREVTYN BETA (Sanofi): ~16.5 million doses were distributed in Europe. Overall 373 reports of suspected side effects; 0 reports of suspected autoimmune encephalitis associated with this vaccine.

In summary, reports of suspected autoimmune encephalitis in the context of COVID-19 vaccination are extremely rare. Importantly, it should be noted that vaccines containing mRNA for production of the spike protein, or those that contain the spike protein itself, cannot be affected by molecular mimicry of anti-NMDA receptor antibodies via NSP8 and NSP9 as these sequences come from a separate region of the virus (see: Induction of CNS autoimmunity by SARS-CoV-2). In the case of vector vaccines, immune reactions against the vector virus could play a separate role.

## Limitations

This study has a number of limitations that should be considered before further studies in this area are attempted. First, we only identified 19 cases of SARS-CoV-2-related autoimmune NMDA receptor encephalitis which included 7 cases from our previous study [45]. Moreover, in some of these cases, the criteria for NMDA receptor encephalitis diagnosis were incomplete or not fully described [47], and most of these studies were heterogeneous in their reporting, particularly with regards to circulating and image-based parameters. Often, neither the test method used to determine COVID-19 positivity (antigen assay, PCR, antibody titre) nor the assay used to determine NMDA receptor antibodies, was stated in the case reports. However, there are differences in sensitivity and specificity depending on the test method used. Three of the cases had autoimmune encephalitis in association with a COVID-19 vaccination [68, 69, 72], four were linked with ovarian teratoma [68, 70, 73, 76] and one with focal epilepsy [67]. Another reason that makes it difficult to draw conclusions regarding these cases is that information is missing concerning how many of these patients had experienced a previous SARS-CoV-2 infection. In such cases, vaccination might represent a second exposure to SARS-CoV-2 antigens, especially for the one who had received the Sinopharm vaccine (non-replicating whole virus). One critical aspect is whether individuals with autoimmune encephalitis following SARS-CoV-2 infection are predisposed to this disease manifestation by an underlying compromised immune system or if it arises due to the actual impact of the SARS-CoV-2 virus on immune function. This raises the question of whether the occurrence of autoimmune encephalitis could be merely a non-specific indication of a pre-existing poor immune system or if it is directly linked to the viral infection. Regrettably, the immune function of the patients included in the case reports was largely unexplored. It should also be noted that we restricted our search to probable or definite NMDA receptor encephalitis cases linked with SARS-CoV-2 infection. However, it is possible that some cases were missed due to causes such as incomplete investigation, loss of records, or decisions not to publish due to difficulties encountered during the unprecedented pandemic situation.

Another limitation of our study concerns the point that we were unable to determine the most critical factors related to disease outcome. This is important as not all of the cases responded positively to the described therapeutic approaches, which mainly involved administration of corticosteroids and IVIg. Further studies are needed to address the above points in order to set standards for improved patient outcomes. This should include screening cases for blood and CSF biomarkers, such as inflammation-related molecules, antineuronal antibodies, in addition to brain imaging, EEG analyses and monitoring of neurological and psychiatric symptoms [47, 48, 110]. We suggest that there should be a consensus so that future studies collect the same complete data as well as patient histories. For SARS-CoV-2 infections, this will be difficult as most people who had the virus were never tested. The usual test for the presence of SARS-CoV-2 nucleocapsid protein antibodies would be required to establish this.

## Conclusions and future perspectives

Although our findings do not support a definitive link between SARS-CoV-2 infection or vaccination and neuronal autoantibody-mediated encephalitis, we hope that this meta-analysis has helped to increase our capacity for differential diagnosis of the neurological and psychiatric symptoms associated with SARS-CoV-2 infections. We anticipate that this will also increase our attentiveness regarding the development, diagnosis and therapeutic management of NMDA receptor encephalitis associated with infections by this virus or by COVID-19 vaccination. This latter point may be important as more 70% of the world population has now received at least one dose of a COVID-19 vaccine [103]. In this study, we identified 19 cases of NMDA receptor encephalitis associated with SARS-CoV-2 infection or vaccination which included 7 cases identified in our previous investigation [45]. In contrast with our previous report which showed favourable responses of all patients to either the first- or second-line treatments, two out of the newly-identified cases showed no improvement and one died. This underlines the need for further research into the pathomechanisms involved in this disorder, combined with standardised procedures put in place to guide predictions of NMDA receptor encephalitis patient outcomes. One way this can be assessed is through the use of clinical, psychological and apparative testing in combination with screening tools for molecular markers, such as specific autoantibody panels. We also suggest the use of artificial intelligence approaches, such as machine and deep learning, to aid in the deconvolution of the expected high dimensionality in the resulting data associated with these approaches, although this approach would first be reasonable if large cohorts are collected.

Even though the World Health Organization has declared that COVID-19 is no longer a global health emergency, vigilance is required to detect the emergence of a potentially novel SARS-CoV-2 variant or a new virus that poses a similar or more deadly threat [111]. In addition, up to 10-30% of COVID-19 cases have resulted in PCS which can include chronic symptoms of fatigue, impairment of cognition, as well as both physical and neurological/neuropsychiatric deficits, as can be observed after other severe infections and medical conditions [112, 113]. Based on these findings and those in the current review, we suggest that further studies are conducted to determine if there is a subgroup of post-COVID syndrome sufferers with autoimmune-mediated dysfunction of the CNS circuitry, as in NMDA receptor encephalitis. It will also be important to incorporate screening procedures such as those described here to increase our understanding of both the acute and chronic effects of viral infection on neurological dysfunction and to aid in our preparedness in case of future coronavirus outbreaks.

### Supplementary information

Supplementary Table

## Footnotes

1 Some seizures can be so severe they lead to excessive uncontrolled discharge of neurons. These discharges can lead to increased metabolism and energy demand in affected brain regions. Repeated and intense seizure episodes can lead to excitotoxic damage of neurons [*Meldrum BS. Prog Brain Res 2002;135:3-11*].

2 Additional safety information for authorised COVID-19 vaccines is available from the European Medicines Agency (EMA) [https://www.ema.europa.eu/en/human-regulatory/overview/public-health-threats/coronavirus-disease-covid-19/covid-19-medicines#safety-information-for-authorised-covid-19-vaccines-section].
