# Supplementary material for: Possible temporal relationship between SARS-CoV-2 infection and anti-NMDA receptor encephalitis: a meta-analysis
Source: Transl Psychiatry. 2024 Mar 8;14:139. doi: 10.1038/s41398-024-02831-0 (PMC10923949; doi:10.1038/s41398-024-02831-0)
Supplement: Supplementary file 1 — Supplementary Table [file 41398_2024_2831_MOESM1_ESM.docx]

**Supplementary Table:** Fulfilment of Graus et al. criteria for NMDA receptor encephalitis [[1](#_ENREF_1)] in the identified case reports.

| **Patient Number** | **1** | **1a** | **1b** | **1c** | **1d** | **1e** | **1f** | **2** | **2a** | **2b** | **3** | **CSF IgG** | **Tera-toma** | **Diagnosis** |
| --- | --- | --- | --- | --- | --- | --- | --- | --- | --- | --- | --- | --- | --- | --- |
| **1** | T | T | T | T | T |  |  | T | T | T | T | **?** | F | **Probable NMDARE** |
| **2** | T | T | T | T |  | T |  | T | T | F | T | T | F | **Definite NMDARE** |
| **3** | T | T |  | T | T | T |  | T | T | T | T | T | T | **Definite NMDARE** |
| **4** | T | T |  | T | T | T |  | T | T | T | T | T | nm | **Definite NMDARE** |
| **5** | T | T |  |  | T | T | T | T | T | T | T | T | T | **Definite NMDARE** |
| **6** |  | T | # | T | T |  |  | T | nm | T | T | **?** | F | unclear* |
| **7** | T | T | T | T |  | T |  | T | T | F | T | **?** | F | **Probable NMDARE** |
| **8** |  | T | T |  |  |  | T | T | T | T | T | T | F | **Definite NMDARE** |
| **9** | T | T | T |  | T | T |  |  | nm | nm | T | **?** | F | unclear* |
| **10** |  | T | T | T |  |  |  | T | T | T | T | T | T | **Definite NMDARE** |
| **11** | T | T | T | T | T | T |  | T | F | T | T | T | F | **Definite NMDARE** |
| **12** | T | T |  | T | T | T |  | T | T | T | T | **?** | F | **Probable NMDARE** |
| **13** | T | T | T |  | T |  | T | T | T | T | T | T | F | **Definite NMDARE** |
| **14** | T | T | T | T |  | T |  | T | T | T | T | T | T | **Definite NMDARE** |
| **15** |  | T |  | T |  | T |  | T | T | T | T | T | nm | **Definite NMDARE** |
| **16** | T | T |  | T | T | T |  | T | T | T | T | T | F | **Definite NMDARE** |
| **17** | T | T | T | T | T |  |  | T | nm | T | T | T | nm | **Definite NMDARE** |
| **18** |  | T |  | T | T |  |  |  | nm | F | T | **?** | F | unclear* |
| **19** | T | T | T | T | T |  |  | T | T | F | T | T | F | **Definite NMDARE** |

F – false; nm – not mentioned; NMDARE – NMDA receptor encephalitis, T – true.

*unclear, as some of the information for the classification was missing in the publication and was not available from the authors upon request.

T Columns 1, 2 and 3 were marked green if the criteria were met (see next page).

**#** Toddler ⇨ The item "Speech dysfunction" could not be reliably assessed.

**?** The authors were contacted but did not reply.

**Diagnostic criteria for NMDA receptor encephalitis proposed by Graus et al. [**[**1**](#_ENREF_1)**]**

| **Probable NMDA receptor encephalitis**  *Diagnosis can be made when all three of the following criteria have been met:*  *1. Rapid onset (less than 3 months) of at least four of the six following major groups of symptoms:*  *1a) Abnormal (psychiatric) behaviour or cognitive dysfunction*  *1b) Speech dysfunction (pressured speech, verbal reduction, mutism)*  *1c) Seizures*  *1d) Movement disorder, dyskinesias, or rigidity/abnormal postures*  *1e) Decreased level of consciousness*  *1f) Autonomic dysfunction or central hypoventilation*  *2. At least one of the following laboratory study results:*  *2a) Abnormal EEG (focal or diffuse slow or disorganised activity, epileptic activity, or extreme delta brush)*  *2b) CSF with pleocytosis or oligoclonal bands*  *3. Reasonable exclusion of other disorders*  Diagnosis can also be made in the presence of three of the above groups of symptoms accompanied by a systemic teratoma. |
| --- |
| **Definite NMDA receptor encephalitis**  Diagnosis can be made in the presence of one or more of the six major groups of symptoms and IgG anti-GluN1 antibodies after reasonable exclusion of other disorders. Antibody testing should include CSF. |

**Reference:**

1. Graus F, Titulaer MJ, Balu R, Benseler S, Bien CG, Cellucci T *et al.* A clinical approach to diagnosis of autoimmune encephalitis. *Lancet Neurol* 2016; **15**(4)**:** 391-404.
